# Multicellular adaptation to electrophysiological perturbations analyzed by deterministic and stochastic bioelectrical models

**DOI:** 10.1038/s41598-024-79087-7

**Published:** 2024-11-11

**Authors:** Javier Cervera, Michael Levin, Salvador Mafe

**Affiliations:** 1 Dept. Termodinàmica, Facultat de Física, Universitat de València, Burjassot, 46100 Spain; 2 Allen Discovery Center, Tufts University, Medford, MA 02155-4243 USA; 3 Wyss Institute for Biologically Inspired Engineering, Harvard University, Boston, USA

**Keywords:** Ion transport, Developmental biology, Computational biology and bioinformatics

## Abstract

Cells can compensate a disruptive change in one ion channel by compensatory changes in other channels. We have simulated the adaptation of a multicellular aggregate of non-excitable cells to the electrophysiological perturbation produced by the external blocking of a cation channel. In the biophysical model employed, we consider that this blocking provokes a cell depolarization that opens a voltage-gated calcium channel, thus allowing toxic Ca^2+^ levels. The cell adaptation to this externally-induced perturbation is ascribed to the multiplicity of channels available to keep the cell membrane potential within a physiological window. We propose that the cell depolarization provokes the upregulated expression of a compensatory channel protein that resets the cell potential to the correct polarized value, which prevents the calcium entry. To this end, we use two different simulation algorithms based on deterministic and stochastic methods. The simulations suggest that because of the local correlations coupling the cell potential to transcription, short-term bioelectrical perturbations can trigger long-term biochemical adaptations to novel stressors in multicellular aggregates. Previous experimental data on planarian flatworms’ adaptation to a barium-containing environment is also discussed.

## Introduction

Cell homeostasis results from the coupling between biochemical, biomechanical, and bioelectrical processes in a dynamic environment. Multicellular aggregates can adapt to changes in the surroundings by means of changes in regulatory networks and gene expression. This adaptation is particularly intriguing in those cases where cells and tissues encounter unfamiliar environments and novel challenges^1–3^, and sudden alterations provoke incremental responses to acquired tolerance^4^. We explore here how cells could handle the possible transcriptional responses to solve electrophysiological challenges in a window time that could not permit a purely random exploration.

One kind of biological problem-solving concerns the navigation of electrophysiological state space by neural and non-neural systems under normal conditions or when exposed to stressors^5–9^. The ability to respond to changes to maintain homeodynamic states is relevant not only on the time-scale of an individual embryo or adult, but also on the evolutionary time-scale of genomic responses to modifications in ion channel and pump genes. Degeneracy and complexity are ubiquitous biological properties^10^ and should play a central role in bioelectrical adaptation, where cells can find solutions to a disruptive change in one ion channel by compensatory changes in other channels. We study a bioelectrically-focused model based on the transcriptional changes that can occur in the ion channels expression after an external perturbation. The basic concepts provide a complementary view to biochemical models^11,12^ and offer new insights into adaptation mechanisms that do not rely on neural networks^13^. Understanding such responses is crucial for basic evolutionary developmental biology and the field of basal cognition^14^. It is especially relevant for biomedical efforts to correct pathological electrophysiological states, because cellular responses to interventions can limit drug efficacy (or, could be exploited to facilitate a new class of drugs that exert long-term repair)^15^.

**Scheme 1:**
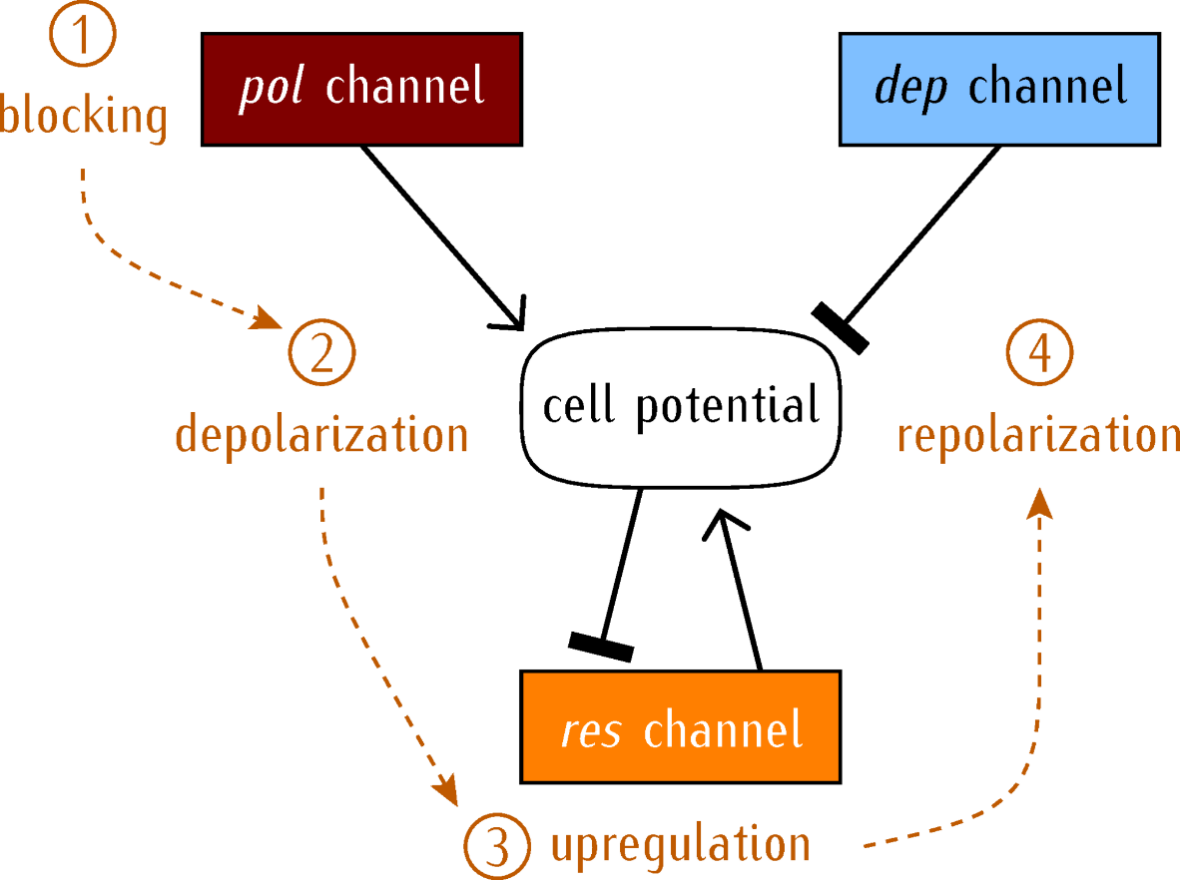
Model summary. The cell response to an external depolarizing stressor. Initially, two counteracting generic channels, the hyperpolarizing (*pol*) channel and the depolarizing (*dep*) channel, establish the physiological cell potential. The blocking of the *pol* channel (1), however, makes the *dep* channel to enforce a relatively depolarized cell potential (2). The resulting depolarization triggers the upregulation of a rescue (*res*) channel (3) which allows cell repolarization to the physiological potential (4). This scheme explicitly accounts for the biological interplay between the genetic and the bioelectric networks and can be extended from the single-cell to the multicellular level by the intercellular gap junctions^16^. The black arrows denote positive regulation while the segments correspond to negative regulation.

The membrane potential is central to bioelectricity because it reflects both the internal cell state and the environmental conditions, including the neighboring cell states^16^. Our adaptation process considers that a potassium channel which regulates the physiological cell potential is blocked, e.g. by an external divalent cation or a blocking molecule. This perturbation causes a short-term cell depolarization. However, because depolarization phenomena are not rare in cell biology, mechanisms opposing this stressor perturbation should be available. Voltage-gated ion channels are characterized by their rapid responses to changes in membrane potential based on modifications of the selective ion conductances^17^. Thus, it is plausible that adaptive re-polarizations be evolutionary encoded by the upregulated transcription of compensatory channels. In our model, we analyze how a rescue channel that conducts outward positive currents could be upregulated to compensate for the electrophysiological stress caused by the externally-induced depolarization (Scheme 1). Experimentally, this case can be relevant to planaria adaptation to a barium environment^4^—a puzzling phenomenon in which planarian flatworms degenerate their heads in the presence of this potassium channel blocker, but then regenerate new functional heads despite its presence.

In non-excitable cells, cell potential windows can be established by multiple ion channels with opposing polarization trends^18^. Thus, the restoration of the physiological potential after blocking must be feasible by changes in the expression of other compensatory channels. Experimentally, the diversity of these channels allows a multitude of self-regulation phenomena based on electro-genetic feedback processes^9,19–22^. This fact makes the *inverse* problem difficult, because different channel conductance combinations should be possible to counter-act cell depolarization. However, the cell would not need to fine-tuning the transcription of every channel protein to establish precise individual conductances, but only to reset *forward* the physiological cell potential (Scheme 1). To this end, we assume here that the membrane potential acts as a *master regulator*^16,21^, both in metabolic and osmotic processes occurring at the *single-cell* level and in developmental and regenerative processes occurring at the *multicellular* level^4,19–21,23^.

Experimentally, bioelectrical cell states can be instructive for biochemical downstream processes^5,24–28^. Multicellular potentials convey *short-term* bioelectrical information to *long-term* transcriptional processes due to the coupling between the electric potentials and the spatio-temporal distributions of signaling ions (e.g., calcium) and molecules (e.g., serotonin)^5^. In model animals, voltage-sensitive dyes evidence that distinct anterior-posterior morphologies can be obtained by changing the axial electric potential^24,25^, which is locally regulated by the ion channel conductances. The intercellular gap junctions, which couple cells into electrical networks^29,30^, can also be important for adaptive phenomena: different planarian head morphologies are observed after junction blocking by octanol^26^. Thus, our model must couple the single-cell potentials at the multicellular aggregate level.

For the sake of generality, we employ two different simulation algorithms that make use of either a deterministic or a stochastic method in the adaptation process. They are based on a well-established electrophysiological fact: the cell *self-regulation* provided by the available multiplicity of ion channels^31^. In particular, the model explicitly accounts for the coupling between the bioelectricity of three channel families and their respective genetic regulator networks, thus offering new insights into adaptation mechanisms that do not rely on neural networks^13^. Also, we pay attention to the intercellular connectivity because the adaptation involves a *community effect* at the multicellular scale^4,26^.

## Results

### Deterministic model

To focus on the adaptive process (see Scheme 1), we have grouped together the model equations used in a final, mathematically-oriented, section. This procedure permits to frame the simulation methods in terms of the biological phenomena studied.

#### Counteracting ion channels define the cell polarization state

**Fig. 1:**
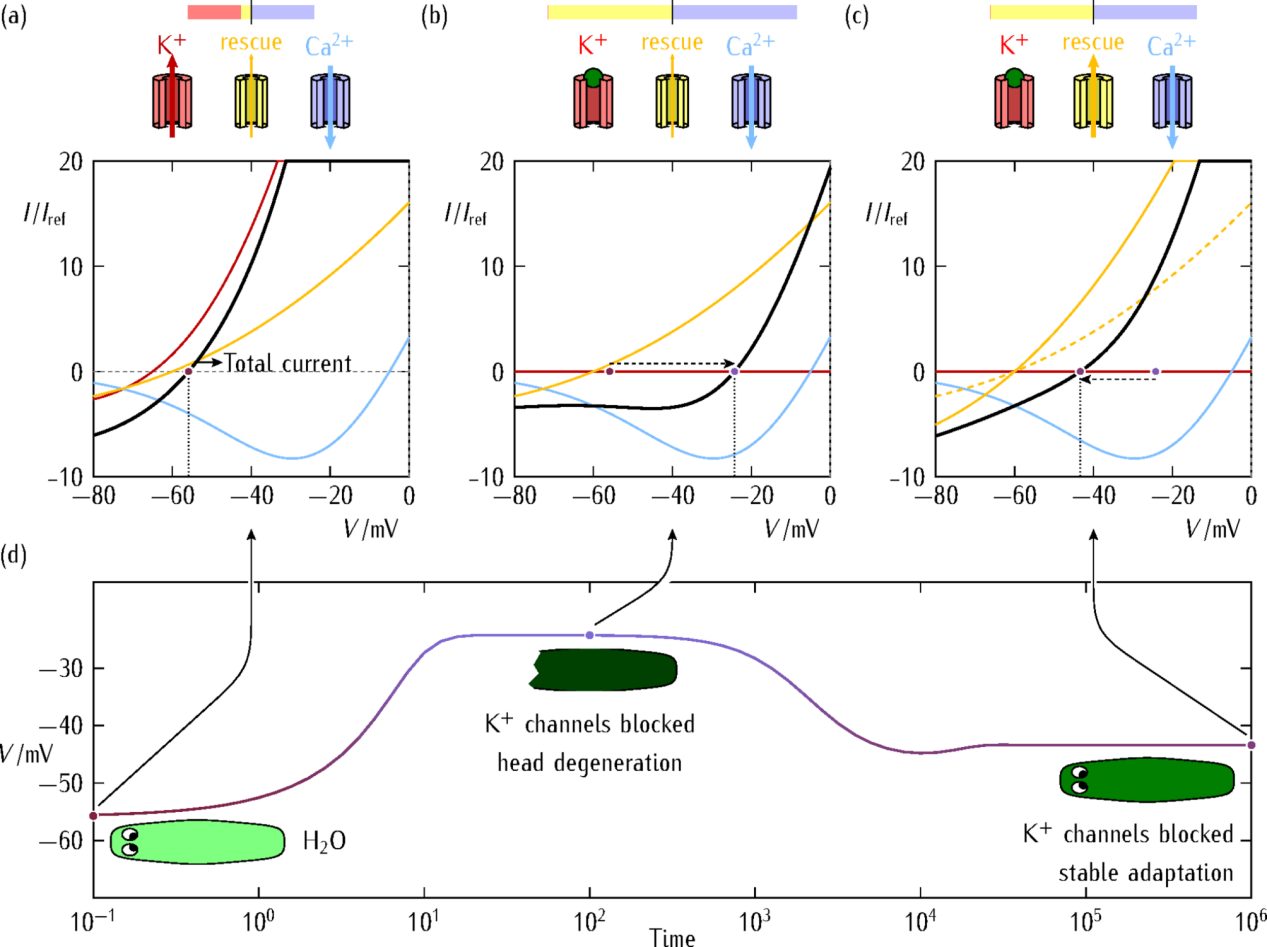
Dominant ion channels and cell polarization states. Schematic of the dominant ion channels and cell polarization states. In each case, the cell potential is regulated by the concerted action of two effective depolarizing (*dep*) and hyperpolarizing (*pol* or *res*) channels of conductances ${G_{{\text{dep}}}}(V)$ and ${G_{{\text{pol}}}}(V)$ or ${G_{{\text{res}}}}(V)$, respectively. The different contributions of these channels to the total current (*top bar* and *colored arrows*) are also shown. **(a)** The *I*−*V* curves of the two opposing channels show the predominantly outward ($I_{{\text{p}\text{o}\text{l}}}^{{}}$, *red curve*) and inward ($I_{{\text{d}\text{e}\text{p}}}^{{}}$, *blue curve*) currents conducted by the potassium and calcium channels, respectively. Before the *pol* channel blocking, these two channels regulate the cell membrane potential, defined by the condition of zero total current (*red point* in the curves). The contribution of the *res* channel to the total current is relatively small initially. The cell is in the physiological polarized state, which is characterized by a cell potential *V* that is close to the equilibrium potential ${E_{\text{p}\text{o}\text{l}}}$ because ${G_{{\text{pol}}}}(V)>{G_{{\text{dep}}}}(V)$. **(b)** When the potassium channel is blocked, the cell potential *V* depolarizes towards ${E_{\text{d}\text{e}\text{p}}}$, where ${E_{\text{p}\text{o}\text{l}}}<V<{E_{\text{d}\text{e}\text{p}}}$, because now ${G_{{\text{pol,blocked}}}}(V)<{G_{{\text{dep}}}}(V)$. This process, which is shown by the *dashed arrow* to the right, marks the onset of the *res* channel, which conducts outward positive currents. **(c)** Cell repolarization can eventually be achieved by the increased expression of the compensatory *res* channel: compare the intermediate (*dashed*) and final (*solid*) curves of this channel. Thus, *V* can repolarize towards the potential ${E_{\text{r}\text{e}\text{s}}}$, which is close to ${E_{\text{p}\text{o}\text{l}}}$, because ${G_{{\text{res}}}}(V)$ increases at the end of the adaptation (*dashed arrow* to the left) due to its negative regulation with *V*. In this figure, we have used the reference current ${I_{\text{r}\text{e}\text{f}}}=1\text{ pA}$ for a reference channel conductance ${G_{\text{r}\text{e}\text{f}}}=0.1\text{ nS}$. **(d)** The time trace of a head cell potential corresponding to the *I−V* curves is shown together with a pictoric view of the model system (*insets*).

The diversity of channel families observed in the transcriptome of model animals^32^ suggests that the compensation of a particular blocked channel should be feasible but anticipates also a complex multidimensional space for modeling. While multiple channels may influence the membrane potential, the fact is that only a relatively small number of them are central to each experimental case^19,20,33–35^. Thus, only a minimal set of generic conductances for the potassium, calcium, and rescue channels (Scheme 1) is considered here. In this way, multiple transmembrane currents are lumped into an operationally tractable but plausible bioelectrical model (Fig. 1).

#### Current (I)−voltage (V) curves

For the sake of concreteness, we consider that the externally-induced depolarization caused by the potassium (*pol*) channel blocking provokes the opening of the calcium (*dep*) channel, increasing the Ca^2+^ level inside the cell and triggering downstream degenerative processes^4^. While this depolarization can proceed through the multicellular aggregate later, we will concentrate first on the single-cell polarized (*pol*) and depolarized (*dep*) states, suggesting how transcriptional changes can eventually occur along the adaptive process.

Figure 1a shows the *I*−*V* curves of two counteracting channels that reproduce the different rectifications observed in potassium and calcium channels. These hyperpolarizing and depolarizing generic channels are characterized by the different rectification parameters explained in the section *Methods*: *biophysical model and simulations*. The two generic channels have *dep* and *pol* conductances that act to establish the equilibrium potentials ${E_{\text{d}\text{e}\text{p}}}$ and ${E_{\text{p}\text{o}\text{l}}}$, which correspond to the zero current conditions${I_{\text{d}\text{e}\text{p}}}=0$ and ${I_{\text{p}\text{o}\text{l}}}=0$, and characterize the *dep* and *pol* cell states, respectively^16,31^. Note that, in this model, the ionic currents and the voltage-gated conductances ${G_{{\text{dep}}}}(V)$ and ${G_{{\text{pol}}}}(V)$ of the *dep* and *pol* channels depend on the cell potential *V*; see the section *Methods*: *biophysical model and simulations* for details.

Experimentally, pairs of opposing voltage-gated channels can be found in neural cells, human cardiomyocytes, pancreatic islets, and biosynthetic tissues, as discussed elsewhere^16^. Figure 1a−c show how a third *res* channel eventually takes the role of the blocked *pol* channel (Scheme 1). The *I*−*V* curve of the *res* channel shows the general qualitative characteristics of an outward-rectifying ion channel that favor cell repolarization, e.g. by a potassium efflux, over a broad voltage window. We make no reference to the channel selectivity here because we associate the adaptive response to the cell membrane repolarization rather than a particular ionic flux. It is conceivable that the compensating *res* channel of voltage-gated conductance ${G_{{\text{res}}}}(V)$ played a similar role to that of the compensated potassium channel that is blocked. Certainly, additional ion pumps and transporters can also be essential for maintaining ionic homeostasis but relatively small changes in the concentrations of the relevant ionic species can still give significant changes in the cell potential when they occur together with lasting changes in the ion channel conductances^16,31^. Thus, it seems plausible to associate the main adaptive counteracting actions to ion channels rather than to pumps. To test further this assumption, it was experimentally checked that some particular channels were upregulated in BaCl_2_-treated animals and that the blocking of these channels by specific agents prevented adaptive transcriptional changes^4^. We must admit, however, that other changes could occur at the level of physiology, not transcription, and it is likely that additional mechanisms of plasticity are also present^4^.

Figure 1a corresponds to the initially unblocked potassium channel. At physiological cell polarizations, the *res* channel current (*yellow curve*) is lower than the absolute values of the individual *pol* and *dep* currents, whose algebraic sum is close the total current (*black curve*). Figure 1b shows the *I*−*V* curves after the blocking of the potassium channel. The resulting decrease in the *pol* channel conductance causes the cell potential depolarization, thus opening the calcium channel and increasing the calcium concentration in the cell. This deviation from homeostasis provokes the bioelectrical stress that elicits a cell response in the form of the *res* channel protein upregulation (Fig. 1c), which provides the adaptive response here. The sequence of Fig. 1a−c corresponds to different experimental times, as shown in Fig. 1d. Typical electrical times $C/{G_{\text{r}\text{e}\text{f}}}$ can be in the range 0.1–1 s for a reference channel conductance ${G_{\text{r}\text{e}\text{f}}}=0.1\text{ nS}$ and a cell capacitance *C* = 10 - 100 pF^16^. On the contrary, transcriptional and translational processes are relatively slow: rate constants of the order of $0.01\text{ - }0.1\text{ min}^{ - 1}$ and degradation rate constants of the order of $0.01\text{ min}^{ - 1}$ suggest time responses of hours^36^. Thus, model times of the order of 10^5^ in Fig. 1d, expressed in $C/{G_{\text{r}\text{e}\text{f}}}$ units, correspond to times in the range 3–30 h.

Because a broad diversity of channels and ion transporters can be found in real cells^31,32^, Fig. 1a−1c can illustrate a rather general mechanism: the *res* channel compensates for the blocking of the *pol* channels and re-establishes the cell polarization state, restoring the cell bioelectrical homeostasis. Note also that a hypothetical blocking of the *res* channel would cause a new cell depolarization from the re-established polarized state because ${G_{{\text{res,blocked}}}}(V)<{G_{{\text{dep}}}}(V)$ in this case. Thus, the model suggests that a specific blocking of the *res* channel could help identifying the particular compensatory channel involved in each adaptation^4^.

#### Cell potential-regulated channel protein transcription

We next describe how the upregulated expression of the *res* channel (Fig. 1c) could be triggered by the blocking-induced cell depolarization (Fig. 1b). Depolarization processes occur e.g. during certain periods of the cell cycle, which is characterized by oscillatory membrane potentials^37^. Thus, cells should have feedback mechanisms to compensate for a depolarizing perturbation and maintain a physiological polarization. The ion channel activity can collectively be influenced at the level of mRNAs by means of the membrane potential^36^, as observed also in neurons^9,38^. If the cell potential can be coupled to multiple channel regulatory networks^16^, it may be plausible that the downstream response to an externally-forced depolarization is an increase in the effective transcription rate of the *res* channel mRNA.

Experimentally, the transcription rate of a channel protein can be regulated by a signaling ion or molecule whose concentration *S* depends on the cell potential^16,36^. Here, different events could be involved in the cell depolarization process. The initial blocking of the potassium channel by the Ba^2+^ ions can initiate this process because of the decreased K^+^ efflux and then the resulting depolarization can open the voltage-gated Ca^2+^ ions causing a calcium influx that may contribute further to depolarization. Thus, it seems plausible that the transcription rates of the *res* channel opposing this depolarization should depend on the cell potential *V*. In the deterministic model, we introduce an effective *negative* regulation for the cell potential-dependent transcription rate of the *res* channel. This approximation lumps a series of intermediate complex contributions from different signaling actors into a phenomenological voltage-dependent rate, which is assumed to be the final outcome of all these contributions.

For the sake of simplicity, we have introduced the effective *negative* regulation for the cell potential-dependent transcription rate of the *res* channel as ${r_{{\text{m,res}}}}(V)=r_{{\text{m},\text{r}\text{e}\text{s}}}^{\circ}/\left[ {1+(S/{S_0})} \right]=r_{{\text{m},\text{r}\text{e}\text{s}}}^{\circ}/\left( {1+{\text{e}^{\upalpha \left| V \right|/{V_\text{T}}}}} \right)$. In this equation, $r_{{\text{m},\text{r}\text{e}\text{s}}}^{\circ}$ and ${S_0}$ are reference values for the transcription rate and the concentration, respectively, *V*_T_ = 27 mV is the thermal potential^36^, and we have introduced α = 3 in the calculations of Fig. 1. Note that cell depolarization is characterized by less negative values of *V* (Fig. 1b), thus decreasing the absolute value $\left| V \right|$ and increasing the transcription rate ${r_{{\text{m,res}}}}(V)$. The resulting upregulated expression of the *res* channel eventually increases its effective conductance, thus repolarizing the cell potential to a physiological value (Fig. 1c). While bioelectrical changes can occur in the range of seconds to minutes, the transcriptional changes associated with the transcription rate increase could be in the range of hours to days, as shown in Fig. 1d^39,40^.

The above phenomenological equation for ${r_{{\text{m,res}}}}(V)$ describes the coupling between the bioelectrical and transcriptional processes in the deterministic model^16^. It converts the exceedingly difficult *inverse* problem of the relationship between genes and cell bioelectricity into the *forward* problem of keeping a master regulator, the cell potential *V*, within physiological levels. Admittedly, multiple complexes and intermediate steps may be involved in the genetic regulatory networks of adaptation. In this context, the effective rate constant ${r_{{\text{m,res}}}}(V)$, which allows increasing the conductance ${G_{{\text{res}}}}(V)$ in Fig. 1c, can be seen as a coarse-graining approximation for the complex connection between the bioelectrical and transcriptional spaces. Note that this adaptation is based on the evolutionary available bioelectrical space of the cell and can occur within its living time.

#### Intercellular connectivity and multicellular simulations

**Fig. 2:**
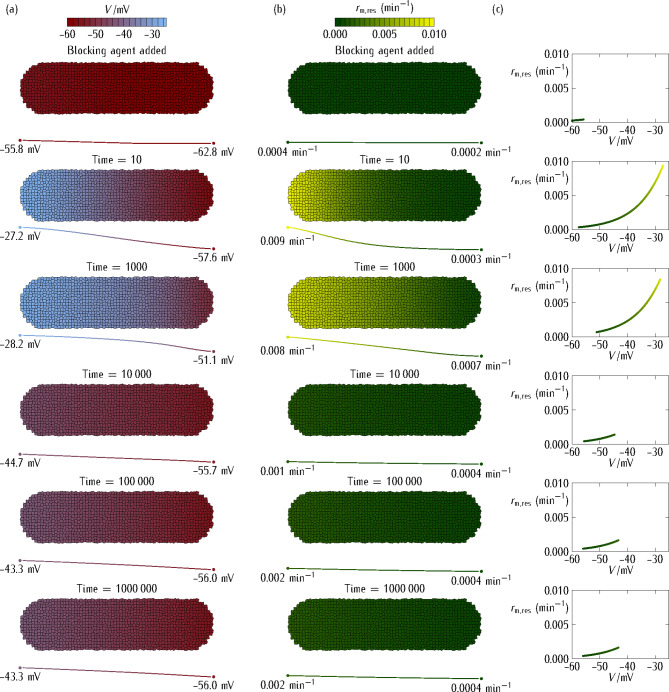
Multicellular aggregate. The multicellular system consists of cells interconnected by gap junctions of individual maximum conductance ${G^\circ}/{G_{\text{r}\text{e}\text{f}}}=0.5$ that permit intercellular currents between neighboring cells. The simulations show the time-dependent values of different bioelectrical magnitudes. **(a)** The multicellular aggregate cell potential *V* (*top inset* bar). **(b)** The multicellular aggregate rate constant ${r_{{\text{m,res}}}}(V)$ of the *res* channel (*top inset* bar). **(c)** The ${r_{{\text{m,res}}}}(V)$* vs. V* deterministic model plot.

We next extend the single-cell polarization states of Fig. 1 to the multicellular system of Fig. 2, which is interconnected by voltage-gated gap junctions of maximum conductance ${G^\circ}$ and average number *n* ≈ 4 of nearest-neighbor cells^36,41^. These intercellular conductances allow the current ${I_{ij}}$ between two neighboring cells *i* and *j*, as explained in the section *Methods*: *biophysical model and simulations*. We consider that the depolarization process is initiated in the *left* (anterior) region of the multicellular aggregate. This would be the case if the ion or molecule that blocks the *pol* channel were not distributed homogeneously in the multicellular system environment. Alternatively, the system could show a *left*/*right* morphological asymmetry reflected by an axial distribution of one of the two dominant channels. The latter experimental possibility is simulated here by introducing an inhomogeneous distribution for the *dep* channel conductance, from $G_{{\text{d}\text{e}\text{p}}}^{\circ}(\textit{left})/{G_{\text{r}\text{e}\text{f}}}=\text{ maximum value}$ to $G_{{\text{d}\text{e}\text{p}}}^{\circ}(\textit{right})/{G_{\text{r}\text{e}\text{f}}}=\text{minimum value}$, according to the position of each cell along the *left*-*right* axis (Fig. 2).

The axial profiles of *V* and ${r_{{\text{m,res}}}}(V)$, averaged over the system cross-section, are also given below each time snapshot. For the sake of concreteness, we consider that cell depolarization is more intense in the *left* than in the *right* region of the multicellular aggregate. To simulate this effect, we introduce a linear profile for the *dep* channel conductance, from $G_{{\text{d}\text{e}\text{p}}}^{\circ}(\textit{left})/{G_{\text{r}\text{e}\text{f}}}=0.8$ to $G_{{\text{d}\text{e}\text{p}}}^{\circ}(\textit{right})/{G_{\text{r}\text{e}\text{f}}}=0.1$, for the central position of each cell along the *left*-*right* axis. The resulting cell potential profile (*bottom inset*), averaged over the system cross-section, is shown for different simulation times (*left scale*). As in Fig. 1, the *pol* channel blocking occurs at time *t* = 0 while the repolarization due to the *res* channel upregulation occurs at times of the order of *t* = 10^5^.

The multicellular system of Fig. 2 consists of a planar monolayer composed of *N* = 1165 cells initially at steady-state cell potentials ${V_i}(t=0)$, *i* = 1,…, *N*. In general, the cell potentials ${V_i}(t)$ evolve according to the balance between: (1) the relative contributions of the *dep*, *pol*, and *res* conductances and (2) the intercellular junction conductances^16^. At time *t* > 0, we set the *pol* conductance to zero and let the aggregate to evolve. Figure 2 shows the multicellular states obtained for the axial profile of cell potentials (Fig. 2a) and *res* channel transcription rates (Fig. 2b) as functions of time. Figure 2c gives the ${r_{{\text{m,res}}}}(V)$*vs. V* plots obtained with the above phenomenological equation for the *res* channel as a function of the cell potentials in each snaphot.

Experimentally, the multicellular patterns can be visualized by voltage-sensitive dyes^4,5,25,42^. Because of the axial profile assumed for the *dep* channel conductance in Fig. 2, the cell depolarization caused by the *pol* channel blocking is stronger in the *left* region than in the *right* region of the aggregate. The extension of the depolarization towards the *right* region, which occurs at intermediate times, is opposed by the repolarizing action of the *res* channel at long times, so that the multicellular adaptation to the perturbation occurs at times *t* > 10^5^.

### Stochastic model

We check now the above deterministic model by introducing a stochastic algorithm where individual cells have a more general capacity for acting on the *res* channel transcription needed to counteract depolarization. In this stochastic model, no phenomenological equation for the relationship between the cell potential *V* and the rate constant ${r_{{\text{m,res}}}}(V)$ of the *res* channel is introduced a priori. In addition, we consider the possibility that those stochastic changes that are not able to avoid a significant long-term depolarization lead to cell death. This additional feature allows a better visualization of the multicellular adaptive process.

#### Stochastic changes in the res channel transcriptional rate

As in the deterministic model, the depolarization causes a significant deviation from the cell bioelectrical homeostasis. However, we assume now that this external stressor makes the cell to relax the initially precise control of the channel expression, thus allowing changes in the regulation of the *res* channel following the *pol* channel blocking. This view implicitly assumes a decreased control of the representational map between cell electrophysiology and transcription^5,36^, which is a plausible assumption under stress conditions.

The cell exploratory dynamics is regulated by the stress-driven feedback between the depolarized and the polarized cell potentials. Thus, we assume that the stochastic change allowed in the *res* channel transcriptional rate ${r_{{\text{m,res}}}}(V)$ is proportional to the difference between the current cell potential and the physiological target potential to be restored. The *res* channel expression is thus iteratively changed to minimize the difference (*error signal*) between these potentials. This adaptive exploration is implemented in the single-cell stochastic algorithm described in the section *Methods*: *biophysical model and simulations*.

#### Multicellular aggregate

**Fig. 3:**
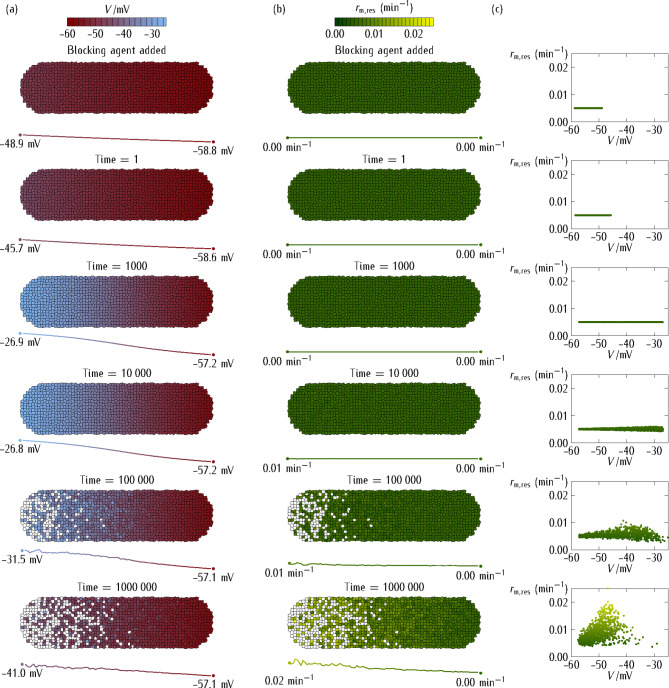
Multicellular aggregate at zero junction conductance. The multicellular system of Fig. 2 in the case of zero junction conductance, ${G^\circ}/{G_{\text{r}\text{e}\text{f}}}=0$ (isolated cells, no intercellular coupling). The simulations show the time-dependent values of different bioelectrical magnitudes. **(a)** The multicellular aggregate cell potential *V* (*top inset* bar). **(b)** The multicellular aggregate rate constant ${r_{{\text{m,res}}}}(V)$ of the *res* channel (*top inset* bar). **(c)** The ${r_{{\text{m,res}}}}(V)$* vs. V* plot for the living cells (*points*).

Figure 3 considers the case of no intercellular coupling (isolated cells). The simulations show the time-dependent multicellular potential (Fig. 3a) and *res* channel rate constant (Fig. 3b). The axial profiles of *V* and ${r_{{\text{m,res}}}}(V)$, averaged over the system cross-section, are also given. Note the significant number of non-viable cells (*white squares*) whose membrane potentials went over the maximum depolarization allowed by the stochastic adaptive process and suffered thus non-physiological depolarizations for a long time. The ${r_{{\text{m,res}}}}(V)$* vs. V* stochastic plot obtained for the living cells (Fig. 3c) suggests a significant decrease of the *res* channel transcription rate with the absolute value of the cell potential *V*, in qualitative agreement with the phenomenological equation assumed in the deterministic model.

The axial profiles of *V* and ${r_{{\text{m,res}}}}(V)$, averaged over the system cross-section, are also given below each time snapshot. The non-viable cells (*white squares*) have local potentials that went over the maximum depolarization allowed during the stochastic adaptive process. As in Fig. 2, we introduce a linear profile for the *dep* channel conductance, from $G_{{\text{d}\text{e}\text{p}}}^{\circ}(\text{h}\text{e}\text{a}\text{d})/{G_{\text{r}\text{e}\text{f}}}=0.8$ to $G_{{\text{d}\text{e}\text{p}}}^{\circ}(\text{t}\text{a}\text{i}\text{l})/{G_{\text{r}\text{e}\text{f}}}=0.1$, for the central position of each cell along the antero-posterior axis.

**Fig. 4:**
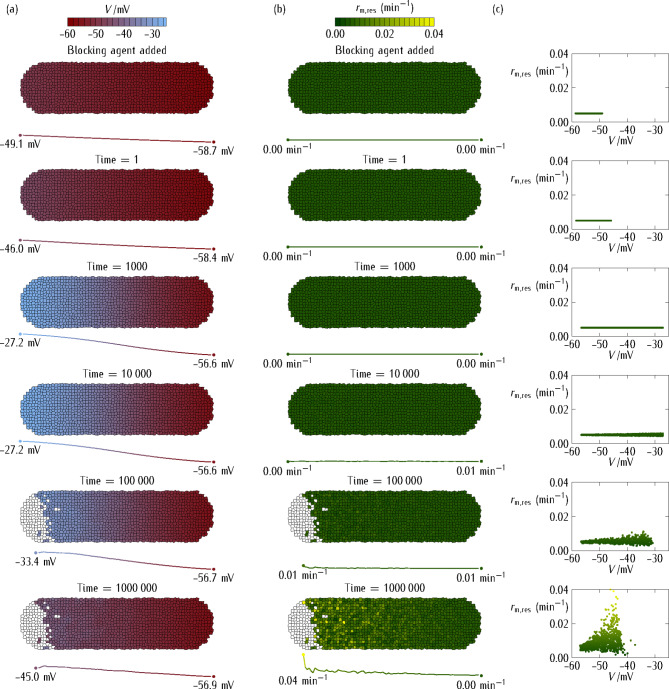
Multicellular aggregate at non-zero junction conductance. The same multicellular aggregate of Fig. 3 except for the junction maximum conductance ${G^\circ}/{G_{\text{r}\text{e}\text{f}}}=0.5$ between neighboring cells. The simulations show the time-dependent values of the different bioelectrical magnitudes. **(a)** The cell potential *V* (*top inset* bar). **(b)** The rate constant ${r_{{\text{m,res}}}}(V)$ of the *res* channel (*top inset* bar). **(c)** The ${r_{{\text{m,res}}}}(V)$* vs. V* plot for the living cells (*points*).

Figure 4 reconsiders the simulations of Fig. 3 for non-zero intercellular coupling. In this case, the adaptation is not a single-cell but a multicellular process in the sense that the *community effect* of the polarized cells in the *right* region counteracts the depolarization process occurring in the *left* region of the aggregate. This effect limits both the spatial extension of the depolarization (Fig. 4a) and the increase in the *res* channel transcription rate (Fig. 4b and c) with respect to those obtained in absence of intercellular coupling (Fig. 3). Note also the relatively low number of non-viable cells obtained in Fig. 4 compared with that of Fig. 3. This result arises from the bioelectrical buffering^16,43^ caused by the polarized potentials of the cells in the *right* region, as they can resist better the depolarization wave caused by the *left* region cells. This bioelectrical effect add to the biochemical buffer effect caused by the now interconnected cell cytoplasm solutions, so that the cells of Fig. 4 behave as a multicellular aggregate to establish and maintain the new adaptive single-cell state of Fig. 1.

To better understand the effects of the intercellular gap junctions in Fig. 4, note that electrically connecting the cells increases the total system capacitance, thus making the multicellular ensemble more resistant to an electrical change (the externally-induced depolarization here). Thus, by avoiding the cell depolarization, the Ca^2+^ influx through voltage-gated calcium channels should be decreased. However, in the hypothetical case (*not* considered here) of *chemical* rather than *electrical* signals, it might be possible to limit the spread of depolarization during excitotoxic cascades to a small local region by *inhibiting* junctional conductance. In this case, junction inhibition could make it more difficult the intercellular diffusion of a toxic agent. Thus, the effects due to the intercellular junctions are context-dependent and it should be understood that the simulation results presented here concern only the *bioelectrical buffering* effect caused by the normally polarized tissue over the depolarized head region.

## Discussion

When we attempt to simulate the cell bioelectrical adaptation to depolarization caused by a potassium channel blockade, the broad repertoire of channels available makes the *inverse problem* difficult for cells: which of the different conductance combinations would counteract the externally-induced depolarization? However, the time required to explore every channel combination would be exceedingly large. Also, the number of possible solutions is severely restricted because channel and gap junction proteins have other physiological functions in addition to regulating the cell potential^5,16,26–29,44^. Thus, most of the potential combinations would not meet all homeostatic requirements and could then be dead-ends for the cell. In the above context, we have assumed that the membrane potential is the master regulation of cell bioelectricity and considered the *forward problem* of restoring its correct value using an effective *res* channel. To this end, we have introduced a voltage-gated feedback for the adaptation that eventually upregulates the expression of the *res* channel, which converts the inverse problem of giving precise values to multiple channel conductances into the forward problem of controlling the physiological cell potential with a reduced number of channels.

We must admit that many error signals and feedbacks, together with other master regulators, might be involved in real cases, but we could expect that the number of cell properties to be controlled should be lower than the total number of available channels. Thus, it can be advantageous to replace the inverse problem of establishing precise individual levels of channel expression by the hopefully simpler problem of identifying the small number of master regulators that control the relevant homeostatic properties. In this scenario, the membrane potential, which can be regulated by a limited number of counteracting channels^16,19,20,33^, could be a choice because of its central role in metabolic, osmotic, developmental, and regenerative processes^5,19,20,27,31,34^.

Here, the following charateristics of the bioelectrical cell potential^5,16,27,34,45–47^ should be emphasized: (1) it shows a high sensitivity to a broad range of chemical, mechanical, and electrical signals, responding rapidly to external changes in the environmemt; (2) it can be tuned by a variety of ions and charged molecules inside and outside the cell; and (3) it allows a multicellular scaling in terms of electric potential patterns that are morphologically instructive. Note also that the membrane potential provides an experimental control of the correlations among channel mRNA levels not only in non-excitable cells^5,36^ but also in neurons^9,38^. In this particular context, the channel and junction proteins may act as the cellular *hardware* while the cell potential could be the *software layer* that fixes the *hardware layer* problem –the blocking of the potassium channel^5,16^. In this way, the bioelectric software enables flexibility to the multicellular aggregate, allowing to respond to environmental stimuli without having to change the hardware.

To characterize the deterministic results further, we have assumed that the depolarization stressor makes the cell to relax the initially precise control of gene expression, thus allowing stochastic changes in the transcription of the *res* channel that permits the adaptation. The transition from tight control to plasticity is a universal adaptation mechanism characteristic of complex systems, from protein structural states to social network responses. The exploration algorithm used leads to results in qualitative agreement with the deterministic model, thus suggesting that the general concepts introduced can be useful.

Experimentally, the simulation results can be relevant to model animals such as planaria. Voltage-sensitive dyes reveal that distinct anterior-posterior planaria morphologies can be obtained by changing the axial electric potential^24,25^, which is regulated by the cell ion channels. In particular, the head degeneration of planaria (*Dugesia japonica*) observed after barium chloride exposure^4^ is ascribed to the Ba^2+^-induced blocking of potassium channels, which provokes the head depolarization^4^. Following this local depolarization, however, long-term changes in the channels expression of the head cells can lead to repolarization and counteraction of the planaria excitotoxic stress. Also, prolonged Ba^2+^ contact eventually results in the regeneration of a new head which maintains form and function despite the presence of barium. Since the neural networks of the brain are mostly destroyed after the first barium chloride exposure, they cannot be the only responsible factor for the adaptation, although they may still influence the subsequent regeneration via additional feedback loops.

In an electrophysiologically-focused description of the above adaptation process^4^, TRPM channels and possibly other alternative cation channels could be upregulated in BaCl_2_-exposed animals: the subsequent blocking of these channels prevents planaria adaptation^4^, as suggested by the model of Fig. 1. Note also that the system response depends on the presence of barium: when a normal water environment is re-established, the unblocking of the potassium channel resets the cell potential to the normal polarized value, thus suppresing the cell stress and then decreasing the rescue channel transcription. As a consequence, it is conceivable that the barium resistance decreased after a sufficiently long period in normal water, as observed experimentally^4^. The above facts suggest that the planaria adaptation could be partly attributed to an electrophysiological exploratory dynamics.

## Conclusion

Using deterministic and stochastic models, we have ascribed the cell adaptation to the *short-term* bioelectrical perturbation that triggers *long-term* downstream biochemical processes because of the local correlation between the electric potential and the distribution of signaling ions and molecules that influence transcription. The basic concepts invoked here provide a complementary view to traditional biochemical models and give new insights into sensing and adaptation mechanisms that do not rely on neural networks. In the biophysical model, the cell does not need to fine-tuning the transcription of every channel protein in order to fix precise conductance values. Instead, it can make use of the cell potential^5,16^ in an error-correcting mechanism that involves a small subset of the total number of cell channels. In principle, these subset of channels can be experimentally identified by external silencing and blocking methods^4,5^. Note also that exploratory adaptations to environmental changes based on modularity^48^ and collectivity^49^ concepts that do not rely exclusively on fine-tuning molecular processes should make easier corrective actions in real systems^50^. Understanding the mechanisms of such error corrections is critical not only to the biomedicine of electrophysiological states, but also to the evolutionary biology of plasticity in development and regeneration as these processes adaptively handle highly novel scenarios^51,52^. In particular, local electric circuits based on ion channels and gap junction networks are crucial in collective cell migration and tissue morphogenesis processes^44,53,54^. Also, ion channels and cell potentials enable active communication and collective responses in bacterial communities^55^.

Here, we implicitly assume that responses to a general class of stressors (cell depolarizing agents here) can be elicited by a novel particular member of that class: while barium might be new to planaria^4^, its effect recalls previous depolarization events that should be counteracted. In this context, the superfamily of voltage-gated ion channels can offer an evolutionary ancient response mechanism^17^. At the single-cell level, it is plausible that the adaptive re-polarization be evolutionary encoded by the upregulated transcription of a rescue channel. At the multicellular level, it is suggested here that the adaptation can also involve a population-level process where the *community effect* of the polarized cells in the *right* region of the aggregate acts to counteract the depolarization process in the *left* region, thus restoring the bioelectrical pattern. Such non-cell-autonomous responses have been seen before in examples of defect repair^56^ and long-range bioelectric signaling in tumor formation^57^, and offer tantalizing targets as biomedical targets.

## Methods

### Deterministic model

**Fig. 5:**
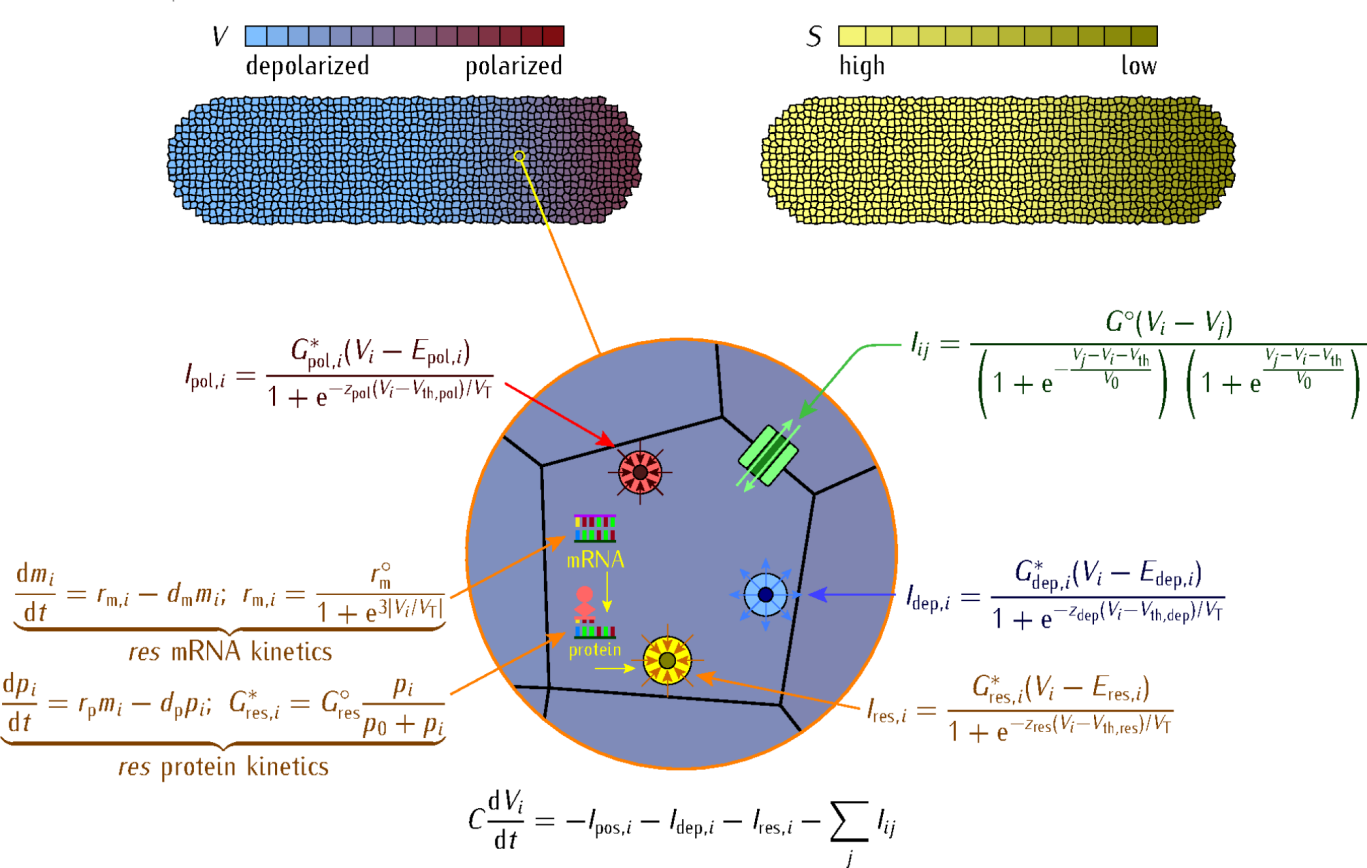
Model equations. The multicellular system of *N* = 1165 cells and the relevant model equations. The scales (*top*) give the cell potential *V* (*left*) and signaling species concentration *S* (*right*) values. The ionic currents ${I_{\text{d}\text{e}\text{p},i}}$ and ${I_{\text{p}\text{o}\text{l},i}}$ of the *i-*th cell correspond to the generic *dep* and *pol* voltage-gated channels of voltage-gated conductances ${G_{{\text{dep,}}i}}=G_{{{\text{dep,}}i}}^{*}/\left\{ {1+\exp \left[ { - z({V_i} - {V_{{\text{th,dep}}}})/{V_\text{T}}} \right]} \right\}$ and ${G_{{\text{pol,}}i}}=G_{{{\text{pol,}}i}}^{*}/\left\{ {1+\exp \left[ {z({V_i} - {V_{{\text{th,pol}}}})/{V_\text{T}}} \right]} \right\}$, with maximum conductances $G_{{\text{d}\text{e}\text{p},i}}^{ * }$and $G_{{\text{p}\text{o}\text{l},i}}^{*}$ and equilibrium potentials ${E_{\text{d}\text{e}\text{p},i}}={E_{\text{d}\text{e}\text{p}}}$ and ${E_{\text{p}\text{o}\text{l},i}}={E_{\text{p}\text{o}\text{l}}}$^31^. The mRNA and protein kinetics of the *dep* and *pol* channels follow similar equations as those of the *res* channel case but they have constant rather than potential-dependent rates in the deterministic model case. The cells are interconnected by an average number of 4 voltage-gated gap junctions of maximum conductance ${G^\circ}$, threshold potential ${V_{\text{t}\text{h}}}$, and reference potential ${V_0}$. This intercellular conductance allows the current ${I_{ij}}$ between two neighboring cells *i* and *j*, where *j* is summed over the nearest-neighbor cells. The bottom equation describes the time-dependent evolution of the cell potentials in the ensemble.

Figure 5 shows the equations of the biophysical model used in the simulations. Note that we do not propose simple equivalent circuits here but explore instead a complete description for the biological interplay between the genetic and the bioelectric networks. This description is extended further from the single-cell to the multicellular scale by the intercellular gap junctions^16^.

Initially, the cells have the isolated membrane potential value and then the multicellular system is allowed to relax to the physiological potential by connecting every cell to its neighbors. To this end, the system is left to evolve for a time ${10^6}C/G_{{\text{r}\text{e}\text{f}}}^{{}}$, where the characteristic bioelectric time $C/G_{{\text{r}\text{e}\text{f}}}^{{}}=0.1\text{ - }1_{{}}^{{}}\text{s}$ for $C=10\text{ - }100_{{}}^{{}}\text{ pF}$ and $G_{{\text{r}\text{e}\text{f}}}^{{}}=0.1_{{}}^{{}}\text{ nS}$. The electrical capacitance *C* characterizes here the individual cell sensitivity to bioelectrical changes. Then, at the simulation time *t =* 0 s the potassium channel is blocked, which is modeled by imposing $G_{{\text{p}\text{o}\text{l}}}^{\circ}=0$, and the system evolves to different bioelectrical states, first the abnormally depolarized state and then the repolarized state, for long times (Fig. 2).

In Fig. 5, the conductances $G_{\text{k}}^{ * }=G_{\text{k}}^{\text{o}}{p_\text{k}}/({p_\text{k}}+{p_{\text{k},0}})$ and the typical parameter values are ${p_{\text{k},0}}=10$, k = *pol* and *dep*, $G_{{\text{p}\text{o}\text{l}}}^{\circ}=G_{{\text{d}\text{e}\text{p}}}^{\circ}=0.1\text{ - }1\text{ nS}$, ${E_{\text{p}\text{o}\text{l}}}= - 65_{{}}^{{}}\text{ mV}$, ${E_{\text{d}\text{e}\text{p}}}= - 5\text{ mV}$, and ${E_{\text{r}\text{e}\text{s}}}= - 60\text{ mV}$, with the threshold potentials ${V_{\text{t}\text{h},\text{p}\text{o}\text{l}}}={V_{\text{t}\text{h},\text{d}\text{e}\text{p}}}={V_{\text{t}\text{h},\text{r}\text{e}\text{s}}}= - {V_\text{T}}= - RT/F= - 27\text{ mV}$for all channels and cells. Here, ${V_\text{T}}=RT/F$ is the thermal potential, where *R* is the gas constant, *T* is the temperature, and *F* is the Faraday constant^16^. The *pol* channel gating charge is *z*_pol_ = 1 and its conductance $G_{{\text{p}\text{o}\text{l}}}^{\circ}/{G_{\text{r}\text{e}\text{f}}}=1.5$ (0 for the case of the *pol* channel blocking). The respective *dep* channel values are *z*_dep_ = 2 and $G_{{\text{d}\text{e}\text{p}}}^{\circ}/{G_{\text{r}\text{e}\text{f}}}=0.8$. For the *res* channel, *z*_res_ = 0.5, with $G_{{\text{r}\text{e}\text{s}}}^{\circ}/{G_{\text{r}\text{e}\text{f}}}=1.5$. In the deterministic model, the genetic regulatory networks are characterized by ${r_{\text{m},\text{k}}}={r_{\text{p},\text{k}}}=0.2\text{ min} ^{ - 1}$ for the mRNA transcription and protein translation rates, respectively, and $d_{{\text{m},\text{k}}}^{{}}=d_{{\text{p},\text{k}}}^{{}}=0.01\text{ min} ^{ - 1}$ for the respective degradation rates, with k = *pol* and *dep*. For the *res* channel, $r_{{\text{m},\text{r}\text{e}\text{s}}}^{\circ}=0.2\text{ min} ^{ - 1}$ (deterministic model) and $r_{{\text{m},\text{r}\text{e}\text{s}}}^{\circ}=0.005\text{ min} ^{ - 1}$ (stochastic simulations), $r_{{\text{p},\text{r}\text{e}\text{s}}}^{\circ}=0.2\text{ min} ^{ - 1}$, and $d_{{\text{m},\text{r}\text{e}\text{s}}}^{{}}=d_{{\text{p},\text{r}\text{e}\text{s}}}^{{}}=0.01\text{ min} ^{ - 1}$. The *res* protein reference concentrations are ${p_{\text{r}\text{e}\text{s},0}}=2$ (deterministic model) and ${p_{\text{r}\text{e}\text{s},0}}=10$ (stochastic simulations). The intercellular conductance can take zero, ${G^\circ}/{G_{\text{r}\text{e}\text{f}}}=0$ (isolated cells), and non-zero (connected cells) values, with *V*_0_ = 10 mV and *V*_th_ = 20 mV. Note that we write all conductance values in terms of a reference conductance ${G_{\text{r}\text{e}\text{f}}}$. The coupling between the bioelectrical and genetic networks of Fig. 5 provides a biophysical scheme based on environmentally-dependent cell states and transitions, including bistability, oscillatory, and synchronization phenomena, that impact on the different gene expression options^16^.

The evolution of the system is calculated from the local equations of Fig. 5 by using a finite-difference scheme. First, maximum variation for the cell potential at every time step is set at ${V_{\textrm{max} }}={10^{ - 3}}\text{ mV}$. Similarly, maximum variations of the *res* channel mRNA and protein concentrations are set at ${m_{\textrm{res,max} }}={m_{\text{r}\text{e}\text{s}}}(t=0)/100$ and ${p_{\text{r}\text{e}\text{s},\textrm{max} }}={p_{\text{r}\text{e}\text{s}}}(t=0)/100$, respectively. For the sake of the numerical control, we consider the minimum and maximum time steps $\Delta {t_{\textrm{min} }}={10^{ - 3}}(C/G_{{\text{r}\text{e}\text{f}}}^{{}})$ and $\Delta {t_{\textrm{max} }}={10^2}(C/G_{{\text{r}\text{e}\text{f}}}^{{}})$, respectively. Then, the time variation of every cell potential *V*_*i*_ is calculated from the equations of Fig. 5 for each simulation time. The time step is $\Delta t=\hbox{min} \left[ {{V_{\textrm{max} }}/(\text{d}{V_i}/\text{d}t),{m_{\textrm{max} }}/(\text{d}{m_{i,\text{k}}}/\text{d}t),{p_{\textrm{max} }}/(\text{d}{p_{i,\text{k}}}/\text{d}t)} \right]$ (k = *pol*, *dep*, *res*) for all *i*, keeping $\Delta {t_{\textrm{min} }} \leqslant \Delta t \leqslant \Delta {t_{\textrm{max} }}$. The time, the cell potentials, and the mRNA and protein concentrations are updated as $t \to t+\Delta t$ and ${V_i} \to {V_i}+(\text{d}{V_i}/\text{d}t)\Delta t$, ${m_{\text{k},i}} \to {m_{\text{k},i}}+(\text{d}{m_{\text{k},i}}/\text{d}t)\Delta t$ and ${p_{\text{k},i}} \to {p_{\text{k},i}}+(\text{d}{p_{\text{k},i}}/\text{d}t)\Delta t$, (k = *pol*, *dep*, *res*), respectively. The calculation proceeds until the desired time is reached. Because typical electrical times $C/{G_{\text{r}\text{e}\text{f}}}$ can be in the range 0.1–1 s for a reference channel conductance ${G_{\text{r}\text{e}\text{f}}}=0.1\text{ nS}$ and a cell capacitance *C* = 10 - 100 pF^16^, model times of the order of 10^5^ in $C/{G_{\text{r}\text{e}\text{f}}}$ units correspond to times in the range 3–30 h.

### Stochastic model

To describe the cell adaptive exploration, we have implemented a stochastic algorithm based on a plausible assumption: the tight control of channel expression should be relaxed under cell stress to permit the adaptation. This algorithm implements and exploratory dynamics based on the stress-driven bioelectrical feedback. The evolution of the systems proceeds similarly as in the deterministic model, i.e. by numerically solving the equations of Fig. 5. However, in this case the transcription rates are changed stochastically every time step ${10^4}C/{G_{\text{r}\text{e}\text{f}}}$ after the blocking of the *pol* channel to simulate the adaptive process. To this end, we introduce the relative potential deviation of cell *i* as ${d_{\text{d}\text{e}\text{v},i}}{\text{ }}={\text{ }}0.2\left| {{V_i} - {V_{\text{t}\text{a}\text{r}\text{g}\text{e}\text{t},i}}} \right|/{V_\text{T}}$, where ${V_i}$ is the cell potential and ${V_{\text{t}\text{a}\text{r}\text{g}\text{e}\text{t},i}}$ is the target physiological potential, which corresponds to the value before the *pol* channel blocking. Then, we generate two random numbers per channel following a uniform distribution of values. The first one (${r_\text{b}}$) is in the range $1 - {b_{\text{n}\text{o}\text{i}\text{s}\text{e}}}{\text{ }} \leqslant _{{}}^{{}}{r_\text{b}}<1+{b_{\text{n}\text{o}\text{i}\text{s}\text{e}}}{\text{ }}$, with ${b_{\text{n}\text{o}\text{i}\text{s}\text{e},i}}=0.02$, and accounts simply for the basal noise. The second one (${r_\text{d}}$) is in the range $1 - {d_{\text{d}\text{e}\text{v},i}}{\text{ }}+{\text{ sgn(}}{V_i} - {V_{\text{t}\text{a}\text{r}\text{g}\text{e}\text{t},i}})\frac{{{d_{\text{d}\text{e}\text{v},i}}}}{{10}} \leqslant _{{}}^{{}}{r_\text{d}}<1+{d_{\text{d}\text{e}\text{v},i}}{\text{ }}+{\text{ sgn(}}{V_i} - {V_{\text{t}\text{a}\text{r}\text{g}\text{e}\text{t},i}})\frac{{{d_{\text{d}\text{e}\text{v},i}}}}{{10}}$ and incorporates a biased noise term because of the small asymmetry provided by the sign (sgn) function. This term results in a biased exploration, according to our assumption that the cell potential should act as a master regulator.

The stochastic algorithm progresses according to the following process. When ${V_i}>{V_{\text{t}\text{a}\text{r}\text{g}\text{e}\text{t},i}}$, the potential ${V_i}$ of cell *i* is depolarized with respect to its target value ${V_{\text{t}\text{a}\text{r}\text{g}\text{e}\text{t},i}}$. Then, we change the *dep* and *res* channel transcription rates by ${r_{\text{m},\text{d}\text{e}\text{p},i}}{\text{ }} \to {r_{\text{m},\text{d}\text{e}\text{p},i}}{\text{ }} \times _{{}}^{{}}{r_\text{b}}$ and ${r_{\text{m},\text{r}\text{e}\text{s},i}}{\text{ }} \to {r_{\text{m},\text{r}\text{e}\text{s},i}}{\text{ }} \times _{{}}^{{}}{r_\text{b}} \times _{{}}^{{}}{r_\text{d}}$, respectively. In this way, the *dep* channel rate changes following the basal noise while the *res* channel rate changes following both the basal noise and biased exploration. On the contrary, when ${V_i}<{V_{\text{t}\text{a}\text{r}\text{g}\text{e}\text{t},i}}$, the cell *i* potential is polarized with respect to its target value. Then, we change the *dep* and *res* channel transcription rates by ${r_{\text{m},\text{d}\text{e}\text{p},i}}{\text{ }} \to {r_{\text{m},\text{d}\text{e}\text{p},i}}{\text{ }} \times _{{}}^{{}}{r_\text{b}} \times _{{}}^{{}}{r_\text{d}}$ and ${r_{\text{m},\text{r}\text{e}\text{s},i}}{\text{ }} \to {r_{\text{m},\text{r}\text{e}\text{s},i}}{\text{ }} \times _{{}}^{{}}{r_\text{b}}$, respectively. In this case, it is the *dep* channel rate that changes following both the basal noise and biased exploration while the *res* channel rate changes following the basal noise only. The small asymmetry in ${r_\text{d}}$ allows the channel rates ${r_{\text{m},\text{k}}}$ to change with time in order to compensate for the difference between the cell potential and the target physiological potential. When ${V_i}>{V_{\text{t}\text{a}\text{r}\text{g}\text{e}\text{t},i}}$ (depolarized cell) the parameter ${r_\text{d}}$ makes ${r_{\text{m},\text{r}\text{e}\text{s},i}}{\text{ }}$to increase in order to repolarize the cell towards ${V_{\text{t}\text{a}\text{r}\text{g}\text{e}\text{t},i}}$. When ${V_i}<{V_{\text{t}\text{a}\text{r}\text{g}\text{e}\text{t},i}}$ (hyperpolarized cell), ${r_\text{d}}$ makes ${r_{\text{m},\text{d}\text{e}\text{p},i}}{\text{ }}$ to increase in order to depolarize the cell towards ${V_{\text{t}\text{a}\text{r}\text{g}\text{e}\text{t},i}}$. This scheme also prevents an overshooting of the above repolarization/depolarization processes. To this end, a maximum value of ${r_{\text{m},\text{k}}}$ should be introduced to prevent an uncontrolled increase. Note that the changes in the *pol* channel rate ${r_{\text{m},\text{p}\text{o}\text{l},i}}{\text{ }}$could not modify the cell state, because this channel is blocked.

Wherever the potential of cell *i* remains deviated from its target potential a value larger than $\left| {{V_i} - {V_{\text{t}\text{a}\text{r}\text{g}\text{e}\text{t},i}}} \right|<\Delta {V_{\text{m}\text{a}\text{x}}}=20\text{ mV}$ for a time period larger than $0.8 \times {10^5}C/{G_{\text{r}\text{e}\text{f}}}$, the cell is assumed to be non-viable and dies. As could be expected, increasing this viability window results in a smaller number of non-viable cells in the simulations.

## Data Availability

The algorithm used here was initially implemented using Python and Javascript as computing languages.For the sake of efficiency and to take advantage of the Template Numerical Toolkit (TNT) and the JAMA/C++ Linear Algebra Package (https://math.nist.gov/tnt/index.html), the code was eventually implemented in C++. The data obtained from the simulations and analysed during the current study are available from the corresponding authors on reasonable request.
